# Supplementary material for: Plant-Produced Viral Nanoparticles as a Functionalized Catalytic Support for Metabolic Engineering
Source: Plants (Basel). 2024 Feb 11;13(4):503. doi: 10.3390/plants13040503 (PMC10893517; doi:10.3390/plants13040503)
Supplement: Supplementary file 1 [file plants-13-00503-s001.zip › plants-2824396-supplementary.pdf]

Supplementary Material

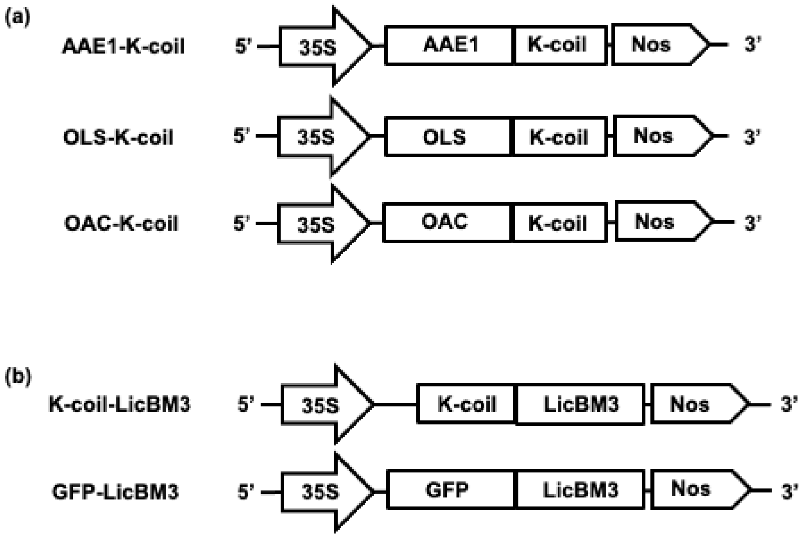

Supplementary Figure S1. Schematic representation of cloned constructs. Transcriptional units for the transient production of enzymes *in planta* were cloned via GoldenBraid cloning system. All transcriptional units are utilizing the P35s promoter from Cauliflower mosaic virus (Prom 35S) and the nopaline synthase terminator from *A. tumefaciens* (Nos-Ter)

(a) Transcriptional units for the production of catalytic enzymes from the cannabinoid synthesis from *C. sativa* AAE1: Acyl activating enzyme 1; OLS: olivetol synthase; OAC: olivetolic acid cyclase.

(b) Transcriptional unit for the production of the thermostable lichenase from *Clostridium thermocellum* (LicBM3) fused with the K-coil-sequence as reporter enzyme.

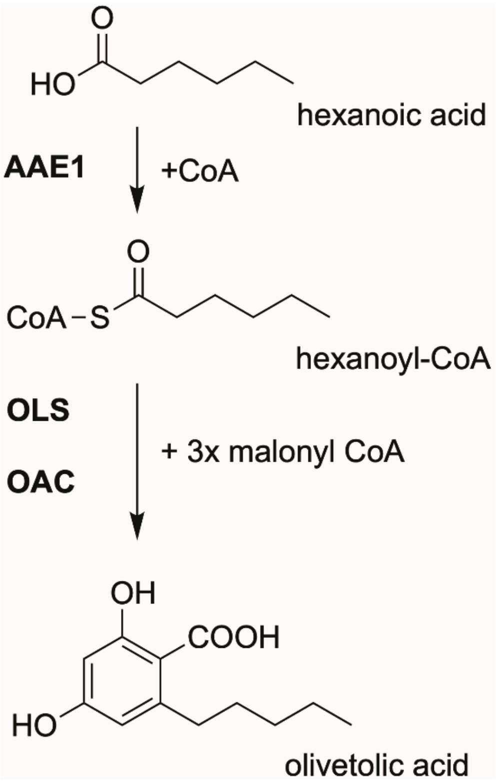

Supplementary Figure S2. Schematic representation of the biosynthetic pathway for production of the cannabinoid precursor olivetolic acid from *C. sativa* (AAE1: acyl activating enzyme 1; OLS: olivetol synthase; OAC: olivetolic acid cyclase).

Supplementary Table S1. List of oligonucleotides used for cloning.

Name	Sequence (5'-3')
TBSVECoilFor	cggaggtaaggaagtgtcagctttggagaaagaggttagtgcacttgaaaaggaggtgtctgcattagaaaaagaagtttctgctcttgagaagtgagagctcttaat
TBSVECoilRev	taagagctctcacttctcaagagcagaaacttctttttctaatgcagacacctccttttcaagtgcactaacctctttctccaaagctgacacttccttacctccgggcc
ECoilPVXFor	ggccgcatgtccaccaaggaagtgtcagctttggagaaagaggttagtgcacttgaaaaggaggtgtctgcattagaaaaagaagtttctgctcttgagaagg
ECoilPVXRev	tcgaccttctcaagagcagaaacttctttttctaatgcagacacctccttttcaagtgcactaacctctttctccaaagctgacacttccttggtggtcatgc
